# Dynamic Interplay between Copper Toxicity and Mitochondrial Dysfunction in Alzheimer’s Disease

**DOI:** 10.3390/life11050386

**Published:** 2021-04-24

**Authors:** Giusy Tassone, Arian Kola, Daniela Valensin, Cecilia Pozzi

**Affiliations:** Department of Biotechnology, Chemistry and Pharmacy–Department of Excellence 2018–2020, University of Siena, Via Aldo Moro 2, 53100 Siena, Italy; giusy.tassone@unisi.it (G.T.); kola2@student.unisi.it (A.K.)

**Keywords:** Alzheimer disease, copper, proteomics, redox proteomics, mitochondria, oxidative stress

## Abstract

Alzheimer’s disease (AD) is a neurodegenerative disorder, affecting millions of people worldwide, a number expected to exponentially increase in the future since no effective treatments are available so far. AD is characterized by severe cognitive dysfunctions associated with neuronal loss and connection disruption, mainly occurring in specific brain areas such as the hippocampus, cerebral cortex, and amygdala, compromising memory, language, reasoning, and social behavior. Proteomics and redox proteomics are powerful techniques used to identify altered proteins and pathways in AD, providing relevant insights on cellular pathways altered in the disease and defining novel targets exploitable for drug development. Here, we review the main results achieved by both -omics techniques, focusing on the changes occurring in AD mitochondria under oxidative stress and upon copper exposure. Relevant information arises by the comparative analysis of these results, evidencing alterations of common mitochondrial proteins, metabolic cycles, and cascades. Our analysis leads to three shared mitochondrial proteins, playing key roles in metabolism, ATP generation, oxidative stress, and apoptosis. Their potential as targets for development of innovative AD treatments is thus suggested. Despite the relevant efforts, no effective drugs against AD have been reported so far; nonetheless, various compounds targeting mitochondria have been proposed and investigated, reporting promising results.

## 1. Introduction

Alzheimer’s disease (AD) is a neurodegenerative disorder representing one of the most common forms of dementia in the elderly. Millions of people worldwide are affected by AD and this number is expected to increase exponentially in the future, since no effective treatments are available so far (Alzheimer’s Association, 2020). AD is characterized by severe cognitive dysfunctions associated with the destruction of neurons and their connections in specific brain areas such as the hippocampus, cerebral cortex, and amygdala, compromising memory, language, reasoning, and social behavior [1]. AD is characterized by two major neuropathological hallmarks, the accumulation of intracellular neurofibrillary tangles and the deposition of extracellular senile plaques. The former consists mainly of hyperphosphorylated microtubule-associated tau protein (tau) whereas the latter primarily contains amyloid-β peptides (Aβ), derived from proteolysis of the amyloid-β precursor protein (APP) [1]. The “amyloid cascade” hypothesis is the main model of AD pathogenesis [2]. Cellular toxicity is due to the aggregated forms of Aβ, in particular Aβ42, inducing neuronal stress and promoting tau hyperphosphorylation [3]. Nonetheless, the failure of the main therapeutic trials based on compounds interdicting with APP processing, points out that targeting the “amyloid cascade” could be counterproductive [4]. Furthermore, this hypothesis does not explain earlier AD phenomena, like aberrant calcium homeostasis, elevated blood levels of cholesterol, altered metabolism of fatty acids and phospholipids, mitochondrial dysfunction, and oxidative stress [5,6,7,8]. Currently, extensive research argues that mitochondrial dysfunction may be induced by Aβ, a hypothesis supporting the “amyloid cascade”, or that it exists independently of Aβ deposition, suggesting a “mitochondrial cascade” as an upstream event in AD insurgence and progression. Therefore, mitochondria seem to initiate or mediate the AD pathologic molecular cascades, supporting their targeting as a new reasonable therapeutic route [9,10]. In AD, mitochondrial dysfunction is also associated with increased oxidative stress levels leading to severe cellular damage [11]. Oxidative stress is also promoted by redox-active metal ions, like Cu(I/II), able to exacerbate the generation of highly reactive oxygen species (ROS) [12].

In the last years proteomic analysis, applied to different in vitro and in vivo AD models, allowed new insights into the main proteins and pathways involved in mitochondrial dysfunction. This research has allowed identification of the biological targets implicated in oxidative stress and to explore the activity of possible new treatments for AD. In this review, we aim to find out the correlation between copper toxicity and oxidative stress by combining mitochondrial proteomic analysis upon Cu-treatments and redox protein modifications (redox proteomics) in AD models. The potential use of compounds targeting the identified proteins are discussed as well.

## 2. Role of Copper in AD

**Figure 1:**
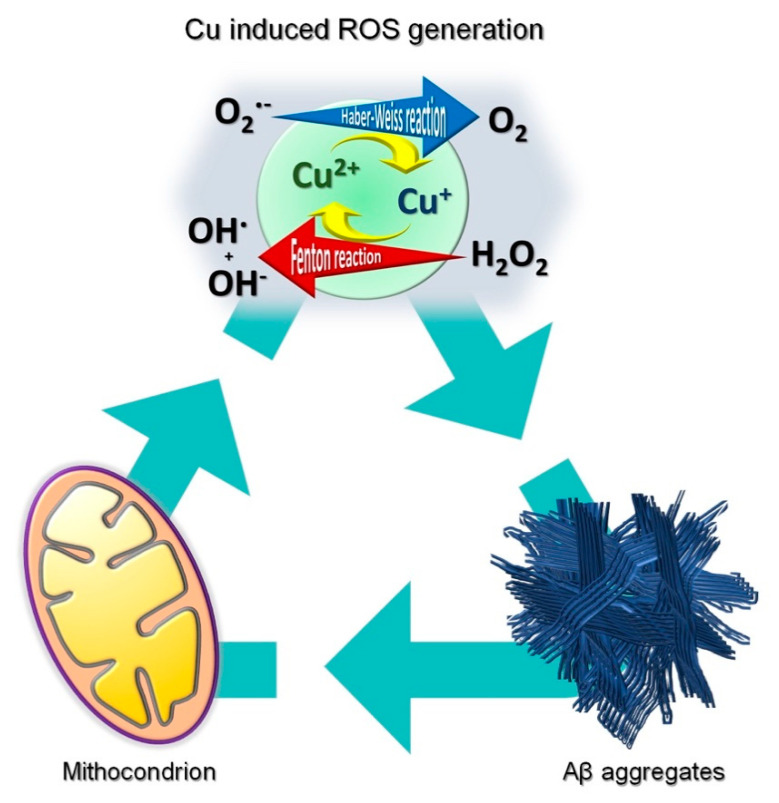
Schematic representation of the interplay between mitochondria activity, copper induced ROS generation and Aβ aggregates.

Copper is an essential trace element found at the highest concentrations in the liver and brain. It acts as a catalytic factor of several enzymes and it is required for normal cellular activity. Under physiological conditions, Cu(I/II) ions are strictly handled by a sophisticated machinery, known as “copper homeostasis”, composed by a huge number of copper chaperones and enzymes preventing its free cellular circulation. Abnormal copper homeostasis is well-documented in AD [13,14] and it is associated with the presence of copper pooling in specific brain areas leading to severe cellular toxicity [15,16,17]. Various forms of dementia are associated with augmented Cu(I/II)-levels in the hippocampus [18,19], and copper-containing Aβ plaques are found in AD brains [20]. The role played by Cu(I/II) ions in AD onset is supported by the correlation between brain copper levels and the prevalence of this disease, as recently shown by the similarities of the AD-affected brain areas and copper toxicity (Figure 1) [21,22]. These are the hippocampus, cerebral cortex, cerebellum, and brainstem leading to dysfunctions in memory, information processing, motor skills, and regulation of autonomous functions.

The central role played by copper in AD is dual, affecting both protein misfolding and oxidative stress (Figure 1) [23]. Its impact on the development of the disease is related to the abilities of Aβ and tau-protein to bind Cu(I) and Cu(II) ions. These copper associations might generate ROS, induce β-sheet structures, and promote protein aggregation in vivo (Figure 1) [12,24]. Two different scenarios have been proposed to explain the effects of increased Cu(I/II) levels on Aβ plaques. In the former, copper leads to an increase in Aβ fibrils and aggregates by promoting β sheet structures. In the latter, the metal triggers a series of inflammatory processes, preventing the clearance of amyloid plaques [25]. In any case, it is well-accepted that copper toxicity impacts on AD progression by targeting homeostatic processes [24,25,26].

In addition to their copper-induced generation, ROS are mainly formed within mitochondria [27] as electron transport chain (ETC, or respiratory chain) byproducts and, if not well-balanced, they might lead to severe mitochondrial and cellular dysfunction [28]. Impairments in mitochondrial electron transfer are correlated with high oxidative stress levels and cognitive dysfunctions. High copper levels may affect mitochondria functions as well by leading to: (i) decreased ATP production; (ii) reduced cytochrome c oxidase activity; (ii) increased permeability of the inner mitochondrial membrane (IMM); (iii) collapse of mitochondrial membrane potential; (iv) increased ADP/ATP ratio; and (v) mitochondrial outer membrane damage [29,30].

## 3. Mitochondrial Proteome Altered upon Copper Exposure in AD Identified by Proteomics Approaches

In the last few years, the development and the growth of -omics platforms yielded to a better understanding of AD, allowing the identification of new biomarkers, protein targets, and pathophysiological mechanisms associated with the disease [31]. Among the -omics science, proteomics is related to the study of the overall proteins present in a cell, tissue or organism and it allows defining protein dysregulation associated with any cellular conditions. A plethora of research teams has applied proteomics to get new advances in the field of AD, with the main aim to improve disease diagnosis and treatments. More than 400 research papers and about 90 scientific reviews have appeared on this topic in the last five years, thus suggesting the relevance of proteomics for AD progress. Proteomics is usually performed by combining two-dimensional gel electrophoresis and mass spectrometry (MS) on digested protein samples derived from (i) tissue disintegration, (ii) organelle isolation, and (iii) protein solubilization or extraction [32].

The proteome investigation of isolated mitochondria in the mouse cortex revealed that long-term low-dose copper treatments lead to abnormal expression of 17 proteins (Table 1 and Table S1) [33]. The most relevant dysregulated proteins in mitochondria are (i) NADH dehydrogenase [ubiquinone] flavoprotein 1 (CI-51kD); (ii) cytochrome b-c1 complex subunit 2 (CIII-s2); (iii) ATP synthase subunit d (ATPase-d); (iv) 75 kDa and (v) 78 kDa glucose-regulated protein (GRP75 and GRP78, respectively). Protein alternative names and reference code are given in Table S2. These five mitochondrial proteins, dysregulated in the brain of Cu-exposed mice, are related to ETC complexes (CIII-s2, CI-51kD and ATPase-d) and apoptosis (GRP75 and GRP78) [33]. Moreover, all of them are involved in AD pathogenesis, supporting the relationship between copper and neurodegeneration.

In particular, GRP75 and GRP78 proteins are members of the heat-shock protein 70 family [35]. They are coupled with mitochondria-associated endoplasmic reticulum membranes (MAM), facilitating the crosstalk between the endoplasmic reticulum (ER) and mitochondria. MAM actively participates in Ca(II)-homeostasis, lipid metabolism, and mitochondrial functions. The connection between AD and MAM alterations is evidenced by mitochondrial dysfunction, Ca(II) dyshomeostasis, high cholesterol levels, and impaired lipid metabolism being common features in the symptomatology of this disease [36,37]. In fact, the expression of GRP75 and the voltage-dependent anion-selective channel protein 1 (VDAC1), forming a functional complex on the mitochondrial membrane, are reduced in the temporal and parietal cortex of AD post-mortem brains Table 1 and Table S1) [38,39]. GRP78 is able to interact with APP in cell culture studies, leading to reduced Aβ40 and Aβ42 secretion in the ER, and thus suggesting a GRP78-mediated protection of APP against β-/γ-secretase [40]. Finally, GRP78 has been recently proposed as a therapeutic target for neurodegenerative diseases [41]. Alterations at the expenses of VDAC1 have been also identified by redox proteomics studies (vide infra Section 4.2).

**Figure 2:**
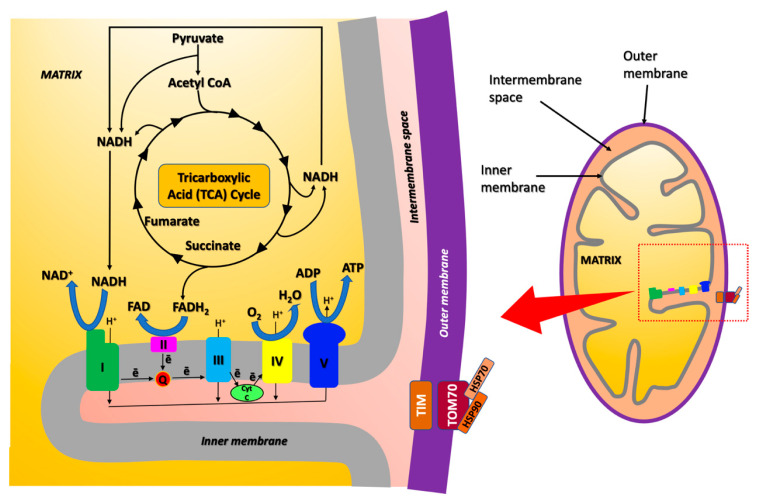
Schematic representation of the ETC protein complexes and of the tricarboxylic acid (TCA) cycle (also known as the Krebs cycle or citric acid cycle) in the mitochondrial matrix.

**Figure 3:**
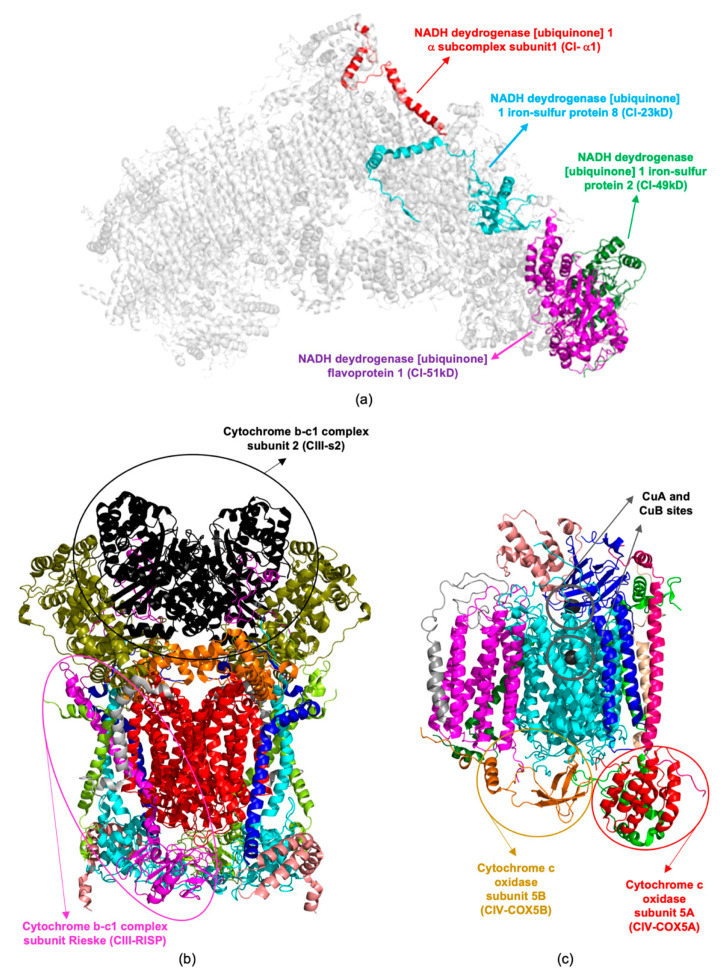
Structures of ETC complex I (**a**), III (**b**), and IV (**c**). Dysregulated and oxidatively modified subunits are evidenced in all complexes. The Protein Data Bank (PDB) codes of the structural models are 5XTD [47], for complex I (human), 5XTE [47], for complex III (human), and 5Z62 [48], for complex IV (human).

CI-51kD, CIII-s2 and ATPase-d are components of the ETC complexes I, III, and V, respectively (Figure 2 and Figure 3). The Kyoto Encyclopedia of Genes and Genomes (KEGG) database (https://www.genome.jp/kegg/, accessed on 10 April 2021) reveals the involvement of these proteins in AD pathways, as also supported by the extensive literature on energy metabolism impairment and mitochondrial dysfunction in AD [42,43,44].

The effect of long-term low-dose Cu(II) exposure was further evaluated on triple-transgenic mouse models of AD (3xTg-AD) [34]. Twenty-four mitochondrial proteins were differentially expressed in hippocampal neurons, among which 14 were upregulated and 10 downregulated in Cu(II)-treated 3xTg-AD mice (Table 1 and Table S3) [34]. Besides the observed perturbations of mitochondrial proteins, a significant alteration of the hippocampal nuclear proteome pointed out the ability of copper to affect the proteins involved in energy metabolism, oxidative stress, DNA damage, nuclear synapses, and apoptosis. Notably, copper-induced effects on protein expression correlate well in 3xTg-AD mice with impaired spatial memory, accumulation of Aβ peptides, and decreased ATP levels.

Mitochondrial ETC dysfunction was already described in several cellular and transgenic mouse models of AD [43,45,46]. However, it is worth considering that Cu-exposure additionally exacerbates the activity of several proteins associated with the mitochondrial ETC complexes (Table 1 and Figure 2). These are NADH dehydrogenase [ubiquinone] 1 α subcomplex subunit 1 (CI-α1 Figure 3a), NADH dehydrogenase [ubiquinone] iron-sulfur proteins 2 (CI-49kD, Figure 3a), and 8 (CI-23kD, Figure 3a), belonging to the complex I, cytochrome b-c1 complex subunit Rieske (CIII-RISP, Figure 3b) of the complex III, cytochrome c oxidase subunit 5A (CIV-COX5A, Figure 3c) and 5B (CIV-COX5B, Figure 3c) of the complex IV, and ATPase-d of the complex V (Figure 2). A brief description of these proteins is given hereafter.

CI-α1 is a component of complex I (Figure 2), essential for its assembly and function [49]. Specific point mutations of this protein are associated with the occurrence of neurological syndrome and a progressive mitochondrial complex I-specific neurodegenerative disease [50,51]. CI-49kD is a fundamental component of the mitochondrial membrane ETC complex I or NADH dehydrogenase. Deeper investigations on CI-49kD have shown that it is important for the complex I machinery, without being essential for its activity [52]. CI-23kD is considered the core subunit of complex I, representing the minimal assembly required for catalysis [53].

CIII-RISP is a component of complex III (also known as ubiquinol-cytochrome c reductase or cytochrome b-c1 complex; Figure 2 and Figure 3b), playing a key role in the Q cycle [54]. CIII-RISP catalyzes the electron transfer from ubiquinol to cytochrome c, with the simultaneous proton translocation across the mitochondrial inner membrane. The impairment of CIII-RISP activity is responsible for mitochondrial dysfunction, associated with AD. The restoring of its expression levels leads to enhancement of central nervous system (CNS) cognitive functions [55].

CIV-COX5A and CIV-COX5B are components of Complex IV, also known as cytochrome c oxidase (CCO), constituted by 13 different subunits (Figure 3c) [56]. CCO is localized at the inner mitochondrial membrane where it accounts for the reduction of molecular oxygen to water. The enzymatic activity of CCO is strictly dependent on the presence of three copper ions populating the CuA and CuB sites (Figure 3c) [57]. Its function in the temporal cortex and hippocampus regions of AD patients is significantly decreased indicating a selective COX-defect in brains [58].

ATPase-d is a component of the ETC complex V, or F_1_F_0_ ATP synthase [59]. This complex is responsible for the oxidative phosphorylation of ADP to generate ATP, exploiting the proton gradient of the ETC as a source of energy. This protein is differentially expressed in the temporal cortex of patients with late-onset AD and its gene expression is associated with the development of Aβ toxicity [60]. Dysfunction of complex V subunits, induced by protein oxidation, was also evidenced by redox proteomics studies (vide infra Section 4.2).

**Figure 4:**
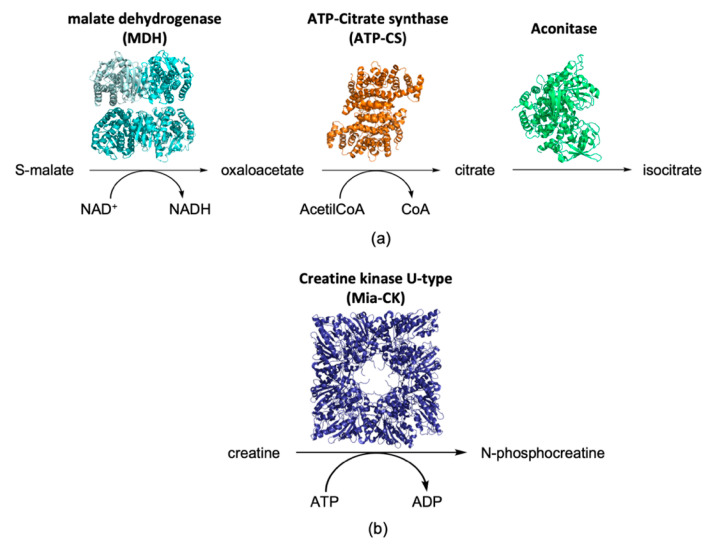
(**a**) Reactions catalyzed by malate dehydrogenase (MDH), ATP-citrate synthase (ATP-CS), and aconitase, in the tricarboxylic acid (TCA) cycle (or Krebs cycle). The PDB codes of the structural models are 4WLF for MDH (human), 5UZQ [63] for ATP-CS (human) and 1C96 [64] for aconitase (*Bos taurus*). (**b**) Reaction catalyzed by creatine kinase U-type (Mia-CK). The PDB code of the structural model is 1QK1 [65].

Beside alterations at the expense of the ETC, Cu(II)-treatments lead to the dysregulation of other metabolic pathways, involving proteins like ATP-citrate synthase (ATP-CS, Figure 4a), malate dehydrogenase (MDH, Figure 4a), pyruvate dehydrogenase E1 component subunit α (PDHE1-A1), and kinase isozyme 2 (PDKII) (Table 1 and Table S1) [34]. These enzymes, participating in the tricarboxylic acid (TCA) cycle (also known as the Krebs cycle or citric acid cycle) and glycolysis, are involved in cellular bioenergetic processes and support energy dysfunctions and cell degeneration in aging [61,62]. Among these enzymes, MDH plays a significant role in mitochondrial dysfunction and it results significantly oxidized in AD brains, as further observed by redox proteomics studies (vide infra, Section 4.2).

Creatine kinase U-type (Mia-CK, Figure 4b) is found in the cristae and intermembrane space of mitochondria, being mainly expressed in the brain. Mia-CK catalyzes the transferring of a phosphate group from ATP to creatine (Cr), generating ADP and PCr, respectively (Figure 4b) [66]. Mia-CK octamers bind to the mitochondrial membranes forming proteolipid complexes with either VDAC1 and ADT/ATP translocase 1 (ANT1), in the intermembrane space, or with ANT alone, in the cristae. ANT1 interaction involves cardiolipin as well, while Ca(II)-dependent complexes are formed with VDAC1. The interplay between mitochondrial and cytosolic CKs plays a central role in energy homeostasis. In AD, the high levels of oxidative stress favor the destabilization of Mia-CK octamers, thus altering the brain energy buffering [67].

## 4. Redox Proteomics to Identify Mitochondrial Altered Proteins

### 4.1. Oxidative and Nitrosative Stress in Mitochondria

Mitochondria are the main source of energy, producing ATP by oxidative phosphorylation, a process that is powered by the electron flow through the highly integrated ETC complexes I-V, spanning across their membrane (Figure 2) [10,68]. Mitochondria are also involved in the regulation of other key cellular processes, including apoptosis, protein synthesis, and oxidation of fatty acids [10,68].

**Figure 5:**
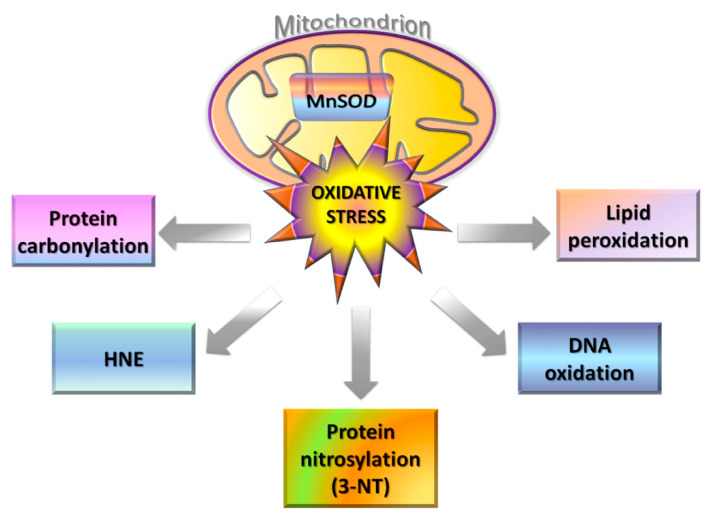
Schematic representation of the oxidative stress effects generated by mitochondria.

On the inner mitochondrial membrane, the ETC complex I relies on both TCA cycle and glycolysis as electron sources (Figure 2) [10,68,69]. The electron flow through the multiple redox-sensitive proteins of the complex I is accompanied by the generation of superoxide radical anions and hydrogen dioxide radicals, promoting oxidative stress (Figure 5) [10,68,69]. Under physiological conditions, superoxide free radicals are neutralized by mitochondrial matrix-resident Mn-dependent superoxide dismutase (MnSOD), which catalyzes their conversion to hydrogen peroxide and molecular oxygen, the former thus degraded by catalases [10,70,71,72]. Despite their high efficiency, peculiar macromolecular packing of catalases in mitochondria decreases the catalytic activity of these enzymes, leading to the accumulation of hydrogen peroxide [10,71,72]. This substance is particularly dangerous since it can diffuse through the mitochondrial membranes, triggering Fenton chemistry reactions in presence of copper or iron ions [10,69,73]. This process leads to the formation of highly reactive species, like hydroxyl anions and hydroxyl free radicals (Figure 1 and Figure 5). Hydroxyl radicals can oxidize proteins by forming carbonyl moieties that alter their structure and function [69,74]. These radicals also react with allylic moieties on the unsaturated fatty acid tails of phospho- and sphingolipids triggering peroxidation processes that generate highly reactive unsaturated aldehydes, as 4-hydroxynonenal (HNE) (Figure 5). These nucleophilic aldehydes could themselves mediate protein modifications, compromising their physiological activities.

A further form of oxidative stress occurs in mitochondria, the nitrosative stress, mediated by nitrogen reactive species (NRS) [69,74,75]. Nitrosative stress is mainly due to nitric oxide synthases (NOSs), enzymes that degrade arginine generating nitric oxide (NO) radicals [75]. Two forms of NOSs are present inside neurons, classified as resident and inducible enzymes. Resident Ca(II)-dependent NOSs are involved in glutamatergic neurotransmission processes, whereas inducible NOSs (i-NOSs) are Ca(II)-independent enzymes, pivotal for brain nitrosative stress. NO radicals produced by these enzymes react with superoxide anions formed within mitochondria, generating nitrogen dioxide (NO_2_) in the presence of CO_2_ [75]. These latter NO_2_ radicals account for nitrosylation of protein tyrosine residues, generating 3-nitrotyrosine (3-NT) (Figure 5). The 3-NT modified proteins are not recognized by tyrosine kinases (TyrKs), blocking phosphorylation-mediated cellular cascades and compromising cell survival [69,71,74,75].

### 4.2. Redox Proteomics in AD

Among the different methods currently developed and applied in the proteomics field, redox proteomics is specifically employed to identify oxidative- and nitrosative-modified proteins [76]. Here, we will review the cornerstones of this technique applied to neurodegenerative disorders and, more specifically, to AD, with a peculiar focus on the identification of mitochondrial oxidized proteins [76,77,78,79]. More details on this technique, mainly developed by Professor Butterfield and his research team, can be found in specific reviews on this topic [76,77,78,80,81]. Redox proteomics relies on the separation of oxidatively modified proteins, which are indexed employing protein-resident carbonyls and 3-NT for protein oxidation and protein-bound HNE for lipid peroxidation. Carbonylation is one of the most frequent oxidative post-translational modifications (PTMs) and the number of carbonyl groups in a protein correlates with its oxidative damage [82,83]. Amino acid carbonylation is due to various processes including direct side chain oxidation, backbone fragmentation, abstraction of H-atoms at Cα, glycation/glycoxidation, and Michael addition reactions (induced by lipid peroxidation byproducts) [83,84,85]. Protein tyrosine nitration, generating 3-NT, is a valid biomarker for protein damage mediated by nitrogen reactive species (RNS) [86]. Indeed, 3-NT is mainly formed by the reaction with peroxynitrite (due to the simultaneous generation of NO and superoxide radicals in presence of CO_2_), but alternative pathways can also occur, including that mediated by myeloperoxidase [76,80]. Lipid peroxidation is among the major sources of free-radical-mediated damages at the expense of membranes, generating also various highly reactive byproducts, including lipid hydroperoxide [76,87,88]. The latter can further decompose to form multiple reactive species, such as HNE, accounting for covalent modification of Cys, Lys and His residues by Michael addition. Two main approaches are currently applied in redox proteomics, gel-based and non-gel based methods, in which specific antibodies are used to detect oxidative- and nitrosative-modified proteins [76,77]. In gel-based methods, protein carbonyls are identified by the treatment with 2,4-dinitrophenylhydrazine (DNPH) to form DNP hydrazone adducts further detected using anti-DNP antibodies. Instead, 3-NT, and 4-HNE modified proteins are directly detected without further derivatization steps, by applying highly specific antibodies recognizing oxidized and nitrosylated targets. On the other hand, in non-gel based methods, modified proteins are identified using enrichment procedures based, for example, on biotin/avidin affinity chromatography for protein carbonyls and 3-NT proteins, or isolation through solid-phase hydrazide beads for HNE-modified proteins/peptides [76]. Individual protein signals of oxidative- and nitrosative-modified proteins are then normalized on the total protein levels and compared across different conditions [76]. In gel-based methods, the oxidized spots of interest are excised from the gel, digested with trypsin and identified using the peptide mass fingerprinting mass spectrometry (PMF-MS) approach, either by matrix-assisted laser desorption ionization (MALDI)-MS or by electrospray ionization (ESI)-MS [76,77,81]. On the other hand, in non-gel based methods, proteins are digested in solution and the peptides separated and identified by nanoflow liquid chromatography separation and automated MS and MS/MS data acquisition and analysis [76,77,81]. Raw MS data collected from these analyses are thus searched against protein databases such as Mascot [89] and Sequest [90], to identify the oxidatively-modified protein targets. Further to the identification, a second aim of redox proteomics is the quantification of the redox levels among different physiological and pathological states. For the quantification of carbonylated proteins, various techniques, including selective reaction monitoring, isobaric tagging, and ^18^O-labelling have been reported so far [91,92]. Redox proteomics represents a powerful resource to recognize and quantify oxidative and nitrosative modified proteins in various diseases, including AD, allowing also the analysis of the pathological cascades occurring in specific cellular compartments, such as mitochondria.

### 4.3. Oxidative Modifications of Mitochondrial Proteins in AD

Redox proteomics has been widely applied to determine oxidatively modified proteins in mitochondria of both in vitro and in vivo AD models and of AD brains [76,77,78,80,81], supporting the hypothesis that both oxidative stress and mitochondrial dysfunction are early events in the insurgence and progression of AD [10,79,93].

The main mitochondrial targets identified by redox proteomics (indexed by carbonylation, nitration, and HNE-derivatization) are proteins primarily involved in energy metabolism, apoptotic processes, antioxidant activity, and ROS scavenging (Table 2 and Figure 2 and Figure 5).

The main oxidatively-modified proteins in AD, taking part in catabolism and generation of ATP within mitochondria, are ATPase subunit α (ATPase-α), VDAC1, and MDH and aconitase, belonging to the TCA cycle [94,95,96,97] (Figure 2 and Figure 4). F-type ATPases, ETC macromolecular complexes embedded in the inner mitochondrial membrane, consist of two structural domains, named F_0_ and F_1_, linked together by a central and a peripheral stalk. F_0_ contains the membrane proton channel, whereas F_1_ is characterized by the extra-membrane catalytic core. ATPase plays a key role in the ETC, coupling the proton gradient established across the inner membrane of mitochondria to promote synthesis and release of ATP (Figure 2). Indeed, the F_1_ ATPase-α catalyzes the oxidative phosphorylation of ADP, generating ATP. This protein has been shown to be HNE-modified in both the early [95] and late AD [94] inferior parietal lobule (IPL) and nitrated in the late AD hippocampus [96] (Table 2). Notably, HNE-modification is coupled with ATPase downregulation in the late-stage AD IPL [94], whereas upregulation is reported in the late AD hippocampus where 3-NT nitrosylation occurs [96] (Table 2). Furthermore, studies on APP/PS1 double knock-in mice models of AD reported ATPase-carbonylation induced by Aβ accumulation [98]. These oxidative modifications of ATPase-α significantly affect its activity, impairing its function [94,95,96,97]. Covalent modifications of ATPase-α are thought to disrupt complex V, impairing its catalytic activity. ATP depletion coupled with changes to other ETC complexes may cause electron leakage from mitochondria, improving ROS production.

**Figure 6:**
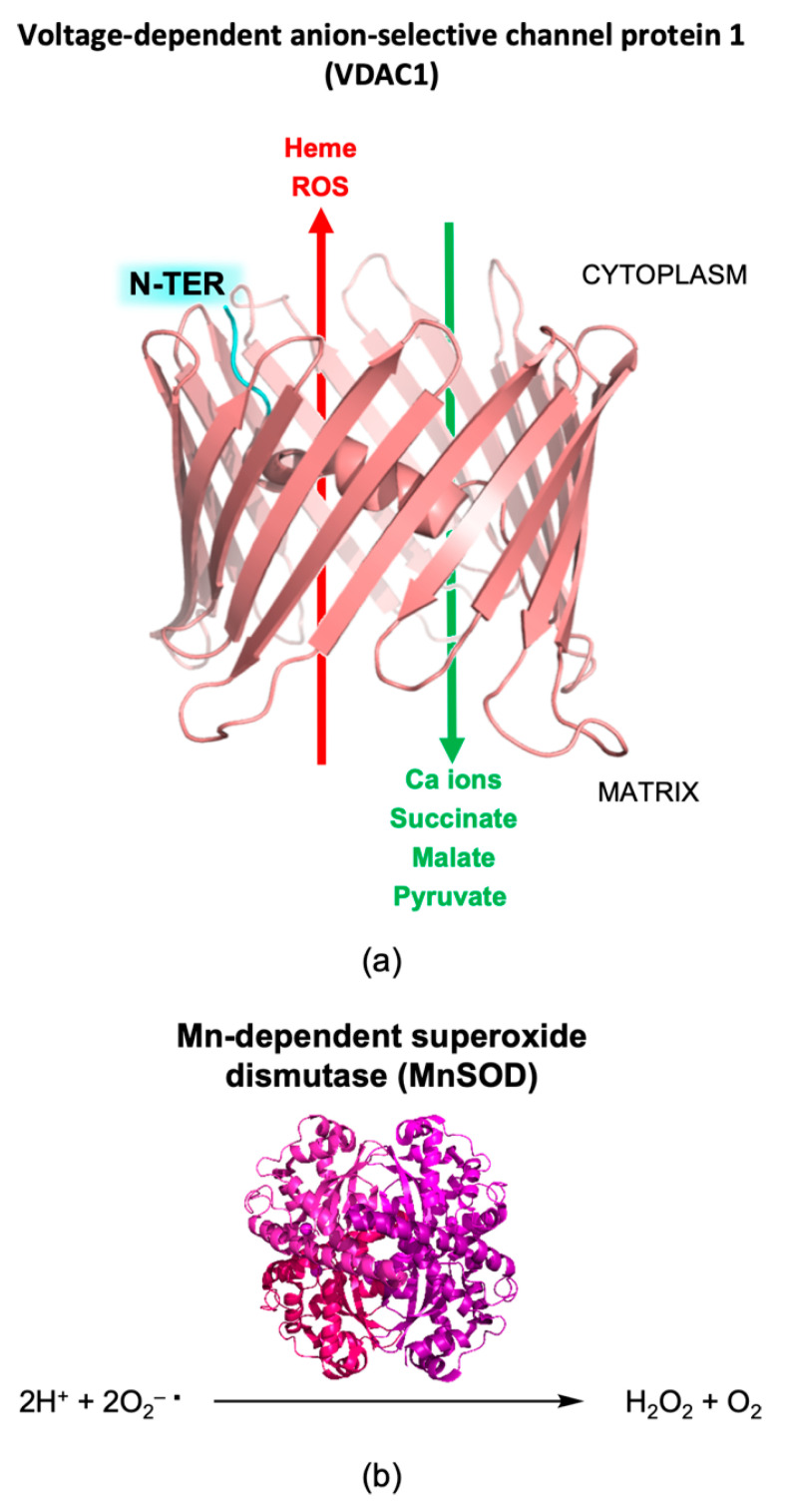
(**a**) Structure of the voltage-dependent anion-selective channel protein 1 (VDAC1, salmon cartoon; the protein N-terminal domain, NTD, is highlighted in cyan). The main substances and ions, trafficking through the channel between the cytoplasm and the mitochondrial matrix, are schematically reported. The PDB code used for the structural model of human VDAC1 is 6G6U. (**b**) Reaction catalyzed by Mn-dependent superoxide dismutase (MnSOD). The PDB code used for the structural model of human MnSOD is 2ADQ [110].

A further mitochondrial protein that undergoes oxidative modifications is VDAC1 [96] (Figure 6a), the outer component of the mitochondrial permeability transition pore (MPTP) [99]. MPTPs are essential for trafficking Ca(II) ions and various metabolites, such as ATP, succinate, malate, and pyruvate, from the cytosol and for the exit of heme and ROS [99,100] (Figure 6a). The gating of the channel is mainly regulated by the VDAC1 N-terminal domain (VDAC1-NTD). Mitochondrial Ca(II) buffering, together with ATP production, are essential processes for synaptic transmission, involving MPTPs in this process [99]. VDAC1 is also involved in apoptotic cascades, through NTD-mediated interactions with various Bcl-2 family members [101,102,103,104,105]. VDAC1 is characterized by a dynamic equilibrium between the monomeric and the oligomeric states. The latter one, induced by the activation of the apoptotic cascade, is responsible for the efflux of cytochrome c from mitochondria, promoting cell death [99]. Upregulation of VDAC1 has been reported in AD brains, showing also its progressive accumulation during the development of the disease, a phenomenon that promotes oligomer formation and apoptosis [106,107]. The VDAC1-NTD protrudes on the cytoplasmic side of the mitochondria outer membrane, where it binds to hexokinase-I (HK-I), an interaction that has a protective role towards apoptosis, as suggested by both in vitro and in vivo evidence [108]. Aβ42 can disrupt this interaction, releasing VDAC1 and triggering its oligomerization together with the release of cytochrome c to the cytosol. This pro-apoptotic cascade was demonstrated in SH-SY5Y cells, as AD cellular models [109]. In the hippocampus of late AD, VDAC1 has been found significantly nitrated (Table 2), a modification that induces apoptotic phenomena [96]. Although the molecular mechanisms triggered by VDAC1 oxidation have not fully elucidated yet, it has been proposed that it alters the structure of the channel inducing its oligomerization and/or promoting HK-1 release [99,109].

The impairment of energy metabolism in mitochondria is also due to oxidative modifications of two enzymes belonging to the TCA cycle—aconitase and MDH [95] (Table 2 and Figure 2 and Figure 4a). The former is an iron-sulfur enzyme that catalyzes the stereo-specific isomerization of citrate to isocitrate via *cis*-aconitate (Figure 4a). The fundamental role played by the 4Fe-4S cluster during the catalysis makes it a redox-sensitive enzyme [111]. Indeed, aconitase undergoes meaningful HNE-oxidation in the late AD hippocampus, modifications that compromise its function by reducing its catalytic activity [94] (Table 2). In vivo studies on triple transgenic AD mice (3xTg-AD mice), evidenced aconitase carbonylation as an early event in the development and progression of the disease [112,113]. The second oxidatively modified enzyme of the TCA cycle is MDH. MDH is an homotetrameric enzyme with four independent active sites, catalyzing the decarboxylation of L-malate to pyruvate through the reduction of NAD(P)^+^ to NAD(P)H [114,115] (Figure 4a). This enzyme accounts also for the malate–aspartate shuttle, linking glycolysis to ETC by transferring NADH to the complex I and promoting ATP production. The activity of MDH increases in an age-dependent manner, supporting a prominent contribution of this enzyme in AD mitochondrial dysfunction [116]. MDH undergoes HNE-oxidation in early AD IPL, a modification that increases its activity probably through conformational changes [95]. In vivo studies on *Caenorhabditis elegans* expressing human Aβ42 and 3xTg-AD mice, evidenced MDH carbonylation in both AD models [112,113]. It is worth noting that in AD mitochondria, oxidative modifications affect both TCA cycle enzymes, leading to opposite effects on their activity. At variance with aconitase, which is inhibited by oxidation, the activity of MDH is significantly improved. The impairment of aconitase and ATP synthase [94] activities in mitochondria is consistent with the hypometabolism peculiar to AD brains [94,95,117,118].

A further protein found oxidatively modified in AD brains is MnSOD, a Mn-dependent homotetrameric enzyme located in the mitochondrial matrix [94,95] (Figure 6b; Table 2 and Table S2). The activity of this enzyme is critical for keeping the cellular oxidative balance, indeed it has been shown that MnSOD knock-out mice die shortly after birth due to increased oxidative stress [119]. The interconnection between ROS and AD is supported by clinical findings showing upregulation of antioxidant enzymes, such as MnSOD, since the early stages of the disease [120,121]. Redox proteomics results on both early and late AD IPL have evidenced that MnSOD is HNE-modified [94,95]. This was also supported by in vivo studies on APP/PS1 double knock-in mice showing MnSOD nitration in AD brains [122,123]. A decreased activity of this enzyme impairs the scavenging of superoxide anions, propagating free radical damage and leading to mitochondrial dysfunction (Figure 5). Under oxidative stress conditions, the scavenging activity of MnSOD is further inhibited by the translocation of the nuclear transcription factor p53 inside mitochondria, suppressing SOD2 gene expression and triggering apoptotic phenomena [124,125,126]. The interplay between MnSOD and p53 has been suggested to fine-tune the cellular response to oxidative stress. As observed for MnSOD, p53 undergoes carbonylation, and HNE and 3-NT oxidation in AD brains, modifications that may affect their interactions, contributing to promote apoptosis and cellular oxidative stress [127,128]. Cellular stress stimuli also promote p53 translocation into the mitochondria outer membrane, where it binds to Bcl-2 and Bcl-xL, releasing Bax and Bak from MPTPs [126]. This cascade of events also induces the release of cytochrome c, a core apoptotic factor. The findings of redox proteomics analyses on oxidatively modified proteins allow the definition of key processes in AD pathogenesis and progression, evidencing the important role played by oxidative stress and mitochondrial dysfunction.

In AD, oxidative stress phenomena have been also traced in the mitochondria of peripheral cells, as lymphocytes [129,130]. Redox proteomics studies have evidenced increased levels of 3-NT, HNE, and carbonylated proteins in mitochondria extracted from the lymphocytes of AD patients, accounting for alterations of energetic, structural, signaling, and antioxidant functions [129,130]. In lymphocyte mitochondria of the early AD stage, TCA enzymes and ATPase are upregulated, whereas a higher activity of ETC complexes II and IV is observed in the late stage of the disease [129,130,131]. The comparison with other AD peripheral cells, such as platelets, highlights different alteration profiles for mitochondrial proteins; indeed, in AD platelets the CCO activity is decreased [132]. These differences suggest the activation of different cellular responses in AD peripheral cells, further modulated during the progression of the disease. A more detailed view of the modifications occurring in peripheral cells and their relationships with the AD stages could lead to the identification of biomarkers, deeply needed for this neurological disorder.

## 5. Main Protein Targets and Pathways Altered in AD by Copper-Induced and Oxidative Modifications

The combined analysis between proteins altered by Cu-exposure and the oxidatively modified proteins in AD brains leads to the determination of common targets and pathways. The first common target identified through these investigations is MDH, belonging to the TCA cycle. The proteomics results on the Cu-exposed brain show the upregulation of MDH [34]. Coherently, an improved activity of the oxidatively modified MDH is evidenced by redox proteomics [95]. Within the TCA cycle, two further enzymes are dysregulated—aconitase and citrate synthase [34,94]. The former is oxidatively-modified in AD brains, an alteration that impairs its enzymatic activity without altering its cellular levels [94]. At the other end, citrate synthase has been found upregulated upon Cu-induced alterations [34]. These three enzymes account for the subsequent steps in the TCA cycle (Figure 2, Figure 3 and Figure 5) supporting the prominent role of its alteration in the development and progression of AD. Furthermore, MDH also plays a role in the malate–aspartate shuttling, linking glycolysis to ETC and promoting NADH production (Figure 2) [114,115]. NADH can be transferred to the complex I, that exploits it to improve ATP production. The connection between TCA and ETC alterations is further suggested by the dysregulation of three complex I subunits, reported upon Cu-exposure by proteomics analysis [33,34]. This finding supports the interplay of these pathways in neurons; indeed, both are altered during AD development and progression.

Further to complex I, proteomics also evidences dysregulations of other mitochondrial ETC proteins belonging to complexes III, IV, and V [33,34]. The alteration of ETC complexes is coherent with the depletion of ATP production and thus with the mitochondria hypometabolic state peculiar of AD. This is particularly evident in the alteration observed at the expenses of complex V subunits both by redox proteomics, showing oxidative modification of ATPase-α, and upon Cu-exposure, reporting downregulation ATPase-d [33,34,94,95,96].

A third protein found altered by both techniques is VADC1 [34,96]. This protein channel, belonging to the MTPT is oxidatively modified in AD brains impairing the trafficking of substances between cytosol and mitochondria [99,100]. VDAC1 oxidative modifications are also connected with apoptotic phenomena, combining various mitochondrial cascades, mainly involving p53, further oxidatively modified in AD brains [101,102,103,104,105,106,107,108,109]. Coherently, altered expression levels of VDAC1 are also observed upon Cu-exposure, reporting its downregulation. Cu-induced dysregulation involves the concomitant alteration of other proteins related to VDAC1, such as Mia-CK and VDAC-2 [34].

## 6. Targeting Mitochondria in AD

The prominent role played by mitochondrial dysfunction in AD insurgence and progression, makes these organelles attractive targets for the development of novel and effective treatments for this neurological disorder. In this review, we discuss the main molecules reported so far to target mitochondrial proteins and the current understanding about their mechanisms of action.

### 6.1. Targeting Mitochondrial ROS Production: Antioxidants

**Figure 7:**
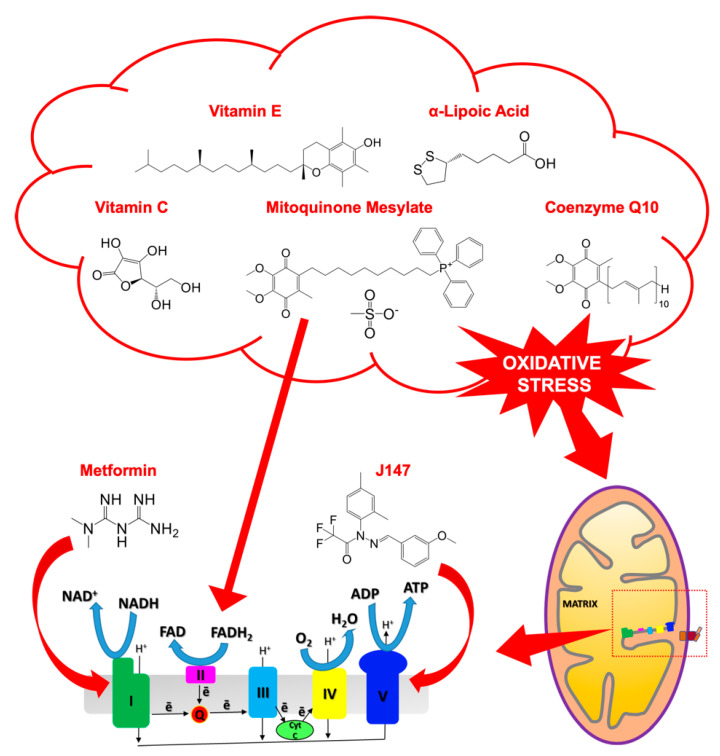
Chemical structures of the therapeutic candidates targeting mitochoindrial ROS production (vitamins C and E, CoQ10, LA, and mitoQ) and ETC (mitoQ, J147, and metformin). The ETC complexes I-V are schematized in the figure, together with the mitochondrion.

Mitochondrial oxidative stress plays an important role in AD onset and progression. An effective strategy to reduce mitochondrial ROS production relies on the administration of antioxidants, such as vitamins C and E, Coenzyme Q10 (CoQ10), α-lipoic acid (LA), and mitoquinone mesylate (mitoQ) (Figure 7) [9,133,134,135].

Vitamin E (Figure 7) refers to two groups of closely related lipophilic compounds— tocotrienols (TCTs) and tocopherols (TCPs)—each having four analogs (named α, β, γ, and δ) [136]. Vitamin E crosses the blood–brain barrier, accumulating at therapeutic levels in the CNS, where it lowers lipid peroxidation, isoprostane levels, and Aβ deposition [137,138]. Several studies demonstrated that lower plasma levels of vitamin E are related to greater risks of AD onset [139]. Even though the therapeutic effects of vitamin E are not fully clarified yet, it was proven that its administration increases plasma levels and diminishes lipoprotein oxidation [140]. Moreover, lower Aβ and tau levels were observed in Tg2576 mice subjected to a vitamin E-supplemented diet [134]. Slower cognitive decline in patients with mild to moderate AD were also reported upon vitamin E treatment [141]. On the other hand, a study on aged male C57BL/6J mice revealed little or no effect on cognitive functions after the administration of vitamin E alone [142]. Notably, meaningful improvements of cognitive and psychomotor impairments were achieved in aged mice through the treatment with vitamin E in combination with CoQ10 [142]. Furthermore, the co-administration of vitamins E and C to AD patients provides consistent positive effects, significantly lowering lipoprotein oxidation [134,140,143].

LA (Figure 7) is a cofactor of both mitochondrial α-ketoglutarate dehydrogenase and pyruvate dehydrogenase and acts as a powerful antioxidant, mediating the recycling of other substances, such as vitamins E and C [144,145]. In AD patients, the principal outcomes obtained with LA are the increased acetylcholine production and the scavenge of lipid peroxidation products, leading to improved cognitive processes and lower mitochondrial oxidative damage [144,145]. The use of LA in combination with other antioxidants (e.g., acetyl-L-carnitine and N-acetyl-L-cysteine) has been extensively investigated, showing improved beneficial effects with respect to LA alone [146,147,148].

CoQ10 (Figure 7) is an essential electron carrier in the mitochondrial ETC and an important antioxidant [149,150,151]. Beside its antioxidant effects in combination with α-tocopherol [142], CoQ10 inhibits apoptosis blocking the activation of the MPTP [152,153]. CoQ10 is also the cofactor of mitochondrial uncoupling proteins that are activated upon its administration, reducing mitochondrial ROS generation [154]. Moreover, reduced Aβ levels and intracellular depositions were observed in aged L235P PS-1 transgenic mice upon CoQ10 treatment [155].

MitoQ (Figure 7) is composed of ubiquinone, an endogenous antioxidant, component of the mitochondrial ETC, covalently bound to triphenylphosphonium (TPP^+^) cation [156]. TPP^+^ drives the ubiquinone moiety to the inner mitochondrial membrane where the ETC complex II reduces it to ubiquinol [157]. MitoQ acts as antioxidant by lowering free radical levels and thus preventing oxidative damage [158,159]. The 3xTg-AD mice treated with a mitoQ-supplemented diet showed higher brain protection towards cognitive decline, Aβ accumulation, astrogliosis, synaptic loss, and caspase activation [160]. A satisfactory pharmacokinetic profile resulted upon mitoQ treatment of AD patients in Phase I clinical trials [154].

### 6.2. Targeting of the Mitochondrial ETC Complexes: J147 and Metformin

J147 is a novel potent compound able to slow down AD progression through neuroprotective effects that result in amelioration of cognition functions. J147 has good medicinal chemical properties and it seems safe and orally active [161]. J147 (Figure 7) is a synthetic derivative of curcumin having a cyclohexyl–bisphenol moiety that confers neurotrophic activity [161]. The effectiveness of J147 was proven in multiple cell culture models mimicking aging and neurodegenerative pathologies [161]. The molecular mechanism of J147 has been recently elucidated, showing its ability to target the mitochondrial ATPase-α [162], acting as an allosteric inhibitor of ATP synthesis [161]. The modulation of ATPase-α by J147 protects neuronal cells, increasing brain-derived neurotrophic factor (BDNF) levels and BDNF-responsive protein expression but lowering oxidative stress and Aβ plaque deposition. Studies in both normal and transgenic AD animals demonstrated that the administration of J147 improved long-term memory and restored cognition in APPswe/PS1ΔE9 mice and in rapidly aging senescence-accelerated dementia (SAMP8) mice [163,164].

The biguanide metformin (Figure 7) is an antihyperglycemic drug used to treat type-2 diabetes, regulating glucose blood levels and liver gluconeogenesis through the stimulation of the AMP-activated protein kinase (AMPK) activity [165]. Metformin also represents a promising drug for AD prophylaxis and therapy, since it prevents hyperinsulinemia, a prompting factor for Aβ plaque formation in AD brains [166]. Furthermore, metformin lowers glucose levels, slowing down glycation end-product formation, inflammation, and oxidative stress [167,168]. The peculiar molecular structure of metformin accounts for its activity as copper-chelating agent [169]. Metformin also interferes with the mitochondrial ETC complex I, inhibiting ATP synthesis and leading to cellular accumulation of AMP, allosterically promoting AMPK activity [170]. In vitro studies showed increased neurogenesis in both human and rodent neurons upon administration of metformin, which stimulates the aPKC–CBP pathway in neural precursors. Improved neurogenesis was also observed in the olfactory bulb and in the hippocampus of adult brains [171]. The reported effects of metformin include amelioration of memory impairment, inhibition of neuronal apoptosis, and reduced Aβ accumulation in the hippocampus [172]. It is worth noting, that studies performed on human neuronal stem cells have demonstrated the neuroprotective effects of metformin against mitochondrial dysfunction caused by Aβ, mediated by the activation of AMPK-dependent pathways [173].

## 7. Conclusions

Proteomics and redox proteomics are powerful techniques to identify altered proteins and pathways in neurological disorders, such as AD. The results achieved through -omics techniques can provide relevant insights on the cellular pathways altered in the disease, leading to the identification of novel targets exploitable for drug development. Here, we have reviewed the main results achieved by both -omics techniques in AD, focusing on the changes occurring in mitochondria under oxidative stress and upon copper exposure. Relevant information arises by the comparative analysis of these results, evidencing alterations of common mitochondrial proteins and also of proteins belonging to the same cycles and cascades. The three commonly identified targets, MDH, ATP synthase, and VDAC-1, play fundamental roles in energy metabolism, oxidative stress, and apoptotic processes, coherently with the increased oxidative damage and the hypometabolic state peculiar of AD brains. These proteins, belonging to the TCA cycles and to the ETC, are suggested as targets exploitable for the development of innovative drugs to treat, or at least to slow down, AD insurgence and progression. Despite the relevant efforts in this field, no effective treatments for AD have been reported so far. Nonetheless, various molecules targeting mitochondrial proteins have been proposed and investigated for this purpose, reporting promising results.

Interesting results arise also from comparative analyses of mitochondrial dysfunctions in neuronal and peripheral cells, evidencing the occurrence of different alterations, variably modulated during the progression of the disease. This information is particularly relevant in view of the identification of potential biomarkers, still lacking for AD and other neurological disorders.

## Figures and Tables

**Table 1:** Summary of dysregulated mitochondrial proteins (protein name and acronym, UniProtKB code and gene name) either in the cortex of copper-exposed mice [33] or in the hippocampus of triple-transgenic mice models of AD (3xTg-AD) [34]. Their expression levels are reported as increased (**⇑**) or decreased (**⇓**). Protein alternative names and reference codes are given in Table S2.

Protein Name	UniProtKB Code (*Mus musculus*)	Gene Name	Expression Level (Cu-Treatment Versus Control)		Reference
			Mice Cortex	3xTg-AD Mice Hippocampus	
NADH dehydrogenase [ubiquinone] flavoprotein 1 (CI-51kD)	Q91YT0	NDUFV1	**⇑**		[33]
Cytochrome b-c1 complex subunit 2 (CIII-s2)	Q9DB77	UQCRC2	**⇑**		[33]
ATP synthase subunit d (ATPase-d)	Q9DCX2	ATP5PD	**⇓**	**⇓**	[33,34]
75 kDa glucose-regulated protein (GRP75)	P38647	HSPA9	**⇓**		[33]
78 kDa glucose-regulated protein (GRP78)	P20029	HSPA5	**⇓**		[33]
NADH dehydrogenase [ubiquinone] 1 α subcomplex subunit 1 (CI-α1)	O35683	NDUFA1		**⇓**	[34]
NADH dehydrogenase [ubiquinone] iron-sulfur protein 2 (CI-49kD)	Q91WD5	NDUFS2		**⇑**	[34]
NADH dehydrogenase [ubiquinone] iron-sulfur protein 8 (CI-23kD)	Q8K3J1	NDUFS8		**⇓**	[34]
Creatine kinase U-type (Mia-CK)	P30275	CKMT1		**⇑**	[34]
ATP-Citrate synthase (ATP-CS)	Q91V92	ACLY		**⇑**	[34]
Malate dehydrogenase (MDH)	P08249	MDH2		**⇑**	[34]
Pyruvate dehydrogenase E1 component subunit α (PDHE1-A1)	P35486	PDHA1		**⇓**	[34]
Pyruvate dehydrogenase (acetyl-transferring) kinase isozyme 2 (PDKII)	Q9JK42	PDK2		**⇑**	[34]
Cytochrome b-c1 complex subunit Rieske (CIII-RISP)	Q9CR68	UQCRFS1		**⇓**	[34]
Cytochrome c oxidase subunit 5A (CIV-COX5A)	P12787	COX5A		**⇓**	[34]
Cytochrome c oxidase subunit 5B (CIV-COX5B)	P19536	COX5B		**⇓**	[34]
Voltage-dependent anion-selective channel protein 1 (VDAC1)	Q60932	VDAC1		**⇓**	[34]
Voltage-dependent anion-selective channel protein 2 (VDAC2)	Q60930	VDAC2		**⇓**	[34]

**Table 2:** Summary of oxidatively modified proteins in AD identified by redox proteomics. The functional state of modified protein and their expression levels are reported as increased (**⇑**), decreased (**⇓**), or unaltered (**⇔**) either in the hippocampus or inferior parietal lobule (IPL). Protein alternative names and reference codes are given in Table S2.

Protein	Oxidative Modification ^1^	Effect on Protein Activity	Expression Level (AD Versus Control)	AD Stage	Brain Region	Reference
Aconitase	HNE	**⇓**	**⇔**	Late	Hippocampus	[94]
MDH	HNE	**⇑**	Not reported	Early	IPL	[95]
ATPase-α	HNE	**⇓**	Not reported	Early	IPL	[95]
	HNE	**⇓**	**⇓**	Late	IPL	[94]
	3-NT	Not reported	**⇑**	Late	Hippocampus	[96]
MnSOD (or SOD2)	HNE	**⇓**	Not reported	Early	IPL	[95]
	HNE	**⇔**	**⇑**	Late	IPL	[94]
VDAC1	3-NT	Not reported	**⇔**	Late	Hippocampus	[96]

^1^ HNE, HNE-modification; 3-NT, nitrosylation.

## Supplementary Materials

The following are available online at https://www.mdpi.com/article/10.3390/life11050386/s1, Table S1: Summary of dysregulated mitochondrial proteins in the cortex of copper-exposed mice. Table S2: Summary of protein alternative names and reference codes for the main mitochondrial targets identified by proteomics and redox proteomics studies. Table S3. Summary of dysregulated mitochondrial proteins in the hippocampus of copper-exposed 3xTg-AD mice.

## Abbreviations

AD, Alzheimer’s disease; ADP, Adenosine diphosphate; AMPK, AMP-activated protein kinase; APP, amyloid-β precursor protein; ATP, Adenosine triphosphate; Aβ, amyloid-β peptides; BDNF, brain-derived neurotrophic factor; CCO, Cytochrome c oxidase; CNS, central nervous system; CoQ10, Coenzyme Q10; ER, endoplasmic reticulum; ETC, electron transport chain; HK-I, hexokinase-I; HNE, 4-hydroxynonenal; IMM, inner mitochondrial membrane; IPL, inferior parietal lobule; KEGG, Kyoto Encyclopedia of Genes and Genomes; LA, α-lipoic acid; MAM, mitochondria-associated endoplasmic reticulum membranes; mitoQ, mitoquinone mesylate; MPTP, mitochondrial permeability transition pore; NADH, Nicotinamide adenine dinucleotide; NO, nitric oxide; NOSs, nitric oxide synthases; NRS, nitrogen reactive species; NTD, N-terminal domain; PDB, Protein Data Bank; ROS, reactive oxygen species; TCA, Tricarboxylic acid; TCPs, tocopherols; TCTs, tocotrienols; TPP^+^, triphenylphosphonium cation; TyrKs, tyrosine kinases; 3-NT, 3-nitrotyrosine; 3xTg-AD, triple-transgenic mouse models.
