# Supplementary material for: Dynamic Interplay between Copper Toxicity and Mitochondrial Dysfunction in Alzheimer’s Disease
Source: Life (Basel). 2021 Apr 24;11(5):386. doi: 10.3390/life11050386 (PMC8146034; doi:10.3390/life11050386)
Supplement: Supplementary file 1 [file life-11-00386-s001.zip › life-1186791-supplementary.pdf]

Dynamic interplay between copper toxicity and mitochondrial dysfunction in Alzheimer's Disease

Giusy Tassone#, Arian Kola#, Daniela Valensin* and Cecilia Pozzi*

Department of Biotechnology, Chemistry and Pharmacy – Department of Excellence 2018-2020, University of Siena, via Aldo Moro 2, Siena, 53100, Italy; giusy.tassone@unisi.it (G.T.), kola2@student.unisi.it (A.K.).

# These authors contributed equally.

* Correspondence: daniela.valensin@unisi.it (D.V.), pozzi4@unisi.it (C.P.); Tel.: +39 0577 232428 (D.V.), +39 0577 232132 (C.P.)

Contents

Table S1 S2

Table S2 S3

Table S3 S5

References S6

Table S1. Summary of dysregulated mitochondrial proteins (name and acronym, UniProtKB code and gene name) in cortex of copper-exposed mice [1]. Their expression levels are reported as increased (⇑) or decreased (⇓). Protein alternative names and reference codes are given in Table S2.

Protein name	UniProtKB code (*Mus musculus*)	Gene name	Expression level (copper treatment versus control)
NADH dehydrogenase [ubiquinone] flavoprotein 1 (CI-51kD)	Q91YT0	NDUFV1	⇑
Cytochrome b-c1 complex subunit 2 (CIII-s2)	Q9DB77	UQCRC2	⇑
ATP synthase subunit d (ATPase-d)	Q9DCX2	ATP5PD	⇓
75 kDa glucose-regulated protein (GRP75)	P38647	HSPA9	⇓
78 kDa glucose-regulated protein (GRP78)	P20029	HSPA5	⇓
Aspartate aminotransferase	P05202	Got2	⇑
Electron transfer flavoprotein subunit α	Q99LC5	ETFA	⇑
Dynein light chain	P63168	DYNLL1	⇑
Macrophage migration inhibitory factor	P34884	MIF	⇑
Dihydropyrimidinase-related protein 2	O08553	DPYSL2	⇑
Vesicle-fusing ATPase	P46460	NSF	⇓
Isocitrate dehydrogenase [NAD] subunit α	Q9D6R2	IDH3A	⇓
Myelin basic protein	P04370	MBP	⇓

Table S2. Summary of protein alternative names and reference codes (UniProtKB and gene name) for the main mitochondrial targets identified by proteomics [1,2] and redox proteomics studies [3–5]. Mouse (*Mus musculus*) and human UniProtKB codes and gene names are given where required.

Protein name	UniProtKB code	Gene name	Alternative protein names
Cytochrome b-c1 complex subunit 2 (CIII-s2)	Q9DB77 (*Mus musculus*) P22695 (human)	UQCRC2 (*Mus musculus*, human)	Complex III subunit 2, Core protein II, Ubiquinol-cytochrome-c reductase complex core protein 2
NADH dehydrogenase [ubiquinone] flavoprotein 1 (CI-51kD)	Q91YT0 (*Mus musculus*) P49821 (human)	NDUFV1 (*Mus musculus*, human)	Complex I-51kD, NADH-ubiquinone oxidoreductase 51 kDa subunit
ATP synthase subunit d (ATPase-d)	Q9DCX2 (*Mus musculus*) O75947 (human)	ATP5PD (*Mus musculus*, human)	ATPase subunit d, ATP synthase peripheral stalk subunit d
75 kDa glucose-regulated protein (GRP75)	P38647 (*Mus musculus*) P38646 (human)	HSPA9 (*Mus musculus*, human)	75 kDa glucose-regulated protein, GRP-75, Heat shock 70 kDa protein 9, Mortalin, Peptide-binding protein 74, PBP74, p66 MOT
78 kDa glucose-regulated protein (GRP78)	P20029(*Mus musculus*) P11021 (human)	HSPA5 (*Mus musculus*, human)	78 kDa glucose-regulated protein1, GRP-78, Binding-immunoglobulin protein1, BiP, Heat shock protein 70 family protein 5, HSP70 family protein 5C, Heat shock protein family A member 5, Immunoglobulin heavy chain-binding protein
NADH dehydrogenase [ubiquinone] 1 α subcomplex subunit 1 (CI-α1)	O35683 (*Mus musculus*) O15239 (human)	NDUFA1 (*Mus musculus*, human)	Complex I-MWFE, CI-MWFE, NADH-ubiquinone oxidoreductase MWFE subunit
NADH dehydrogenase [ubiquinone] iron-sulfur protein 2 (CI-49kD)	O75306 (*Mus musculus*) O15239 (human)	NDUFS2 (*Mus musculus*, human)	Complex I-49kD, CI-49kD, NADH-ubiquinone oxidoreductase 49 kDa subunit
NADH dehydrogenase [ubiquinone] iron-sulfur protein 8 (CI-23kD)	Q8K3J1 (*Mus musculus*) O00217 (human)	NDUFS8 (*Mus musculus*, human)	Complex I-23kD, CI-23kD, NADH-ubiquinone oxidoreductase 23 kDa subunit
Cytochrome b-c1 complex subunit Rieske (CIII-RISP)	Q9CR68 (*Mus musculus*) P47985 (human)	UQCRFS1 (*Mus musculus*, human)	Complex III subunit 5, Cytochrome b-c1 complex subunit 5, Rieske iron-sulfur protein, RISP, Rieske protein UQCRFS1, Ubiquinol-cytochrome c reductase iron-sulfur subunit
Cytochrome c oxidase subunit 5A (CIV-COX5A)	P12787 (*Mus musculus*) P47985 (human)	COX5A (*Mus musculus*, human)	Cytochrome c oxidase polypeptide Va
Cytochrome c oxidase subunit 5B (CIV-COX5B)	P19536 (*Mus musculus*) P10606 (human)	COX5B (*Mus musculus*, human)	Cytochrome c oxidase polypeptide Vb
ATP-Citrate synthase (ATP-CS)	Q91V92 (*Mus musculus*) P53396 (human)	ACLY (*Mus musculus*, human)	ATP-citrate (pro-S-)-lyase, Citrate cleavage enzyme
Malate dehydrogenase (MDH)	P08249 (*Mus musculus*) P40926 (human)	MDH2 (*Mus musculus*, human)	Cytosolic malate dehydrogenase
Pyruvate dehydrogenase E1 component subunit α (PDHE1-A1)	P35486 (*Mus musculus*) P08559 (human)	PDHA1 (*Mus musculus*, human)	PDHE1-A type I
Pyruvate dehydrogenase (acetyl-transferring) kinase isozyme 2 (PDKII)	Q9JK42 (*Mus musculus*) Q15119 (human)	PDK2 (*Mus musculus*, human)	Pyruvate dehydrogenase kinase isoform 2, PDH kinase 2

Creatine kinase U-type (Mia-CK)	P30275 (*Mus musculus*)<br>C9JT96 (human)	CKMT1 (*Mus musculus*)<br>CKMT1A (human)	Acidic-type mitochondrial creatine kinase, Mia-CK, Ubiquitous mitochondrial creatine kinase, U-MtCK
Voltage-dependent anion-selective channel protein 1 (VDAC1)	Q60932 (*Mus musculus*)<br>P21796 (human)	VDAC1 (*Mus musculus*, human)	VDAC-1, VDAC1, Outer mitochondrial membrane protein porin 1, Plasmalemmal porin, Porin 31HL, Porin 31HM
Voltage-dependent anion-selective channel protein 2 (VDAC2)	Q60930 (*Mus musculus*)<br>P45880 (human)	VDAC2 (*Mus musculus*, human)	VDAC-2, VDAC2, Outer mitochondrial membrane protein porin 2
ADT/ATP translocase 1 (ANT1)	P48962 (*Mus musculus*)<br>P12235 (human)	Slc25a4 (*Mus musculus*, human)	ADP,ATP carrier protein 1, ADP,ATP carrier protein, heart/skeletal muscle isoform T1 Adenine nucleotide translocator 1, ANT 1, Solute carrier family 25 member 4
ATP synthase subunit α (ATPase-α)	P25705 (human)	ATP5F1A (human)	ATP synthase F1 subunit α
Aconitate hydratase (Aconitase)	Q99798 (human)	ACO2 (human)	Aconitase, Citrate hydro-lyase
Superoxide dismutase [Mn] (MnSOD)	P04179 (human)	SOD2 (human)	

Table S3. Summary of dysregulated mitochondrial proteins (protein name, UniProtKB code and gene name) in the hippocampus of copper-exposed 3xTg-AD mice [2]. Their expression levels are reported as increased (⇑) or decreased (⇓). Protein alternative names and reference codes are given in Table S2.

**Protein name**	**UniProtKB code (*Mus musculus*)**	**Gene name**	Expression level (copper treatment versus control)
NADH dehydrogenase [ubiquinone] 1 α subcomplex subunit 1 (CI-α1)	O35683	NDUFA1	⇓
NADH dehydrogenase [ubiquinone] iron-sulfur protein 2 (CI-49kD)	Q91WD5	NDUFS2	⇑
NADH dehydrogenase [ubiquinone] iron-sulfur protein 8 (CI-23kD)	Q8K3J1	NDUFS8	⇓
Creatine kinase U-type (Mia-CK)	P30275	CKMT1	⇑
ATP-Citrate synthase (ATP-CS)	Q91V92	ACLY	⇑
Malate dehydrogenase (MDH)	P08249	MDH2	⇑
Pyruvate dehydrogenase E1 component subunit α (PDHE1-A1)	P35486	PDHA1	⇓
Pyruvate dehydrogenase (acetyl-transferring) kinase isozyme 2 (PDKII)	Q9JK42	PDK2	⇑
Cytochrome b-c1 complex subunit Rieske (CIII-RISP)	Q9CR68	UQCRFS1	⇓
Cytochrome c oxidase subunit 5A (CIV-COX5A)	P12787	COX5A	⇓
Cytochrome c oxidase subunit 5B (CIV-COX5B)	P19536	COX5B	⇓
ATP synthase subunit d (ATPase-d)	Q9DCX2	ATP5PD	⇓
Voltage-dependent anion-selective channel protein 1 (VDAC1)	Q60932	VDAC1	⇓
Voltage-dependent anion-selective channel protein 2 (VDAC2)	Q60930	VDAC2	⇓
Elongation factor 1-α 1	P10126	EEF1A1	⇑
Vesicle-fusing ATPase	P46460	NSF	⇑
Actin, aortic smooth muscle	P62737	ACTA2	⇑
Actin, cytoplasmatic 1	P60710	ACTB	⇑
Dihydropyrimidinase-related protein 2	O08553	DPYSL2	⇑
Mitogen-activated protein kinase 1	P63085	MAPK1	⇑
Flotillin-1	O08917	FLOT1	⇑
α-enolase	P17182	ENO1	⇑
LIM and SH3 domain protein 1	Q61792	LASP1	⇑
Septin-6	Q9R1T4	SEPTIN6	⇓

References

1. Lin, X.; Wei, G.; Huang, Z.; Qu, Z.; Huang, X.; Xu, H.; Liu, J.; Zhuang, Z.; Yang, X. Mitochondrial Proteomic Alterations Caused by Long-Term Low-Dose Copper Exposure in Mouse Cortex. *Toxicology Letters* **2016**, *263*, 16–25, doi:10.1016/j.toxlet.2016.10.009.

2. Yu, H.; Wang, D.; Zou, L.; Zhang, Z.; Xu, H.; Zhu, F.; Ren, X.; Xu, B.; Yuan, J.; Liu, J.; et al. Proteomic Alterations of Brain Subcellular Organelles Caused by Low-Dose Copper Exposure: Implication for Alzheimer's Disease. *Arch Toxicol* **2018**, *92*, 1363–1382, doi:10.1007/s00204-018-2163-6.

3. Perluigi, M.; Sultana, R.; Cenini, G.; Domenico, F.D.; Memo, M.; Pierce, W.M.; Coccia, R.; Butterfield, D.A. Redox Proteomics Identification of 4-Hydroxynonenal-Modified Brain Proteins in Alzheimer's Disease: Role of Lipid Peroxidation in Alzheimer's Disease Pathogenesis. *PROTEOMICS – Clinical Applications* **2009**, *3*, 682–693, doi:https://doi.org/10.1002/prca.200800161.

4. Reed, T.T.; Pierce, W.M.; Markesbery, W.R.; Butterfield, D.A. Proteomic Identification of HNE-Bound Proteins in Early Alzheimer Disease: Insights into the Role of Lipid Peroxidation in the Progression of AD. *Brain Research* **2009**, *1274*, 66–76, doi:10.1016/j.brainres.2009.04.009.

5. Sultana, R.; Poon, H.F.; Cai, J.; Pierce, W.M.; Merchant, M.; Klein, J.B.; Markesbery, W.R.; Butterfield, D.A. Identification of Nitrated Proteins in Alzheimer's Disease Brain Using a Redox Proteomics Approach. *Neurobiology of Disease* **2006**, *22*, 76–87, doi:10.1016/j.nbd.2005.10.004.
